# Advancing Interventional Cardiology in Sub-Saharan Africa: The Role of Artificial Intelligence

**DOI:** 10.1016/j.jscai.2025.103791

**Published:** 2025-08-21

**Authors:** Michael A. Negussie, Chernet T. Mengistie, Mikiyas G. Teferi, Fitsum A. Gemechu, Biruk T. Mengistie, Helina K. Teklehaimanot, Amanuel M. Haile

**Affiliations:** School of Medicine, College of Health Sciences, Addis Ababa University, Addis Ababa, Ethiopia

**Keywords:** clinical care, interventional cardiology, policy, sub-saharan Africa

## Introduction

Sub-Saharan Africa (SSA) faces a rapidly increasing burden of cardiovascular disease, yet interventional cardiology services remain significantly limited, primarily accessible only in major urban centers within a few countries.^1^ This disparity severely impacts patient outcomes, especially for time-sensitive conditions such as ST-elevation myocardial infarction (STEMI), for which timely intervention is critical to survival.^2^ While artificial intelligence (AI) offers considerable potential for reducing these health care disparities, effective implementation in SSA must consider practical constraints including limited infrastructure, scarce financial resources, and shortages of health care professionals.^3^ This viewpoint presents a strategic approach to using AI tailored explicitly to these regional challenges, highlighting feasible solutions, barriers, and practical implementation steps.

## Strategic use case: AI-enabled mobile electrocardiogram for STEMI triage

A practical and impactful initiative is the deployment of AI-powered mobile electrocardiogram (ECG) devices specifically designed for rural health care settings. These handheld AI-integrated ECG platforms enable community health workers in primary care facilities to quickly screen patients for acute coronary syndromes and transmit the data directly to specialists in urban cardiology centers. Similar technology has already shown promise in Kenya and Ethiopia, successfully identifying previously undetected conditions such as atrial fibrillation and myocardial infarction with clinically acceptable accuracy.^4^^,^^5^

Implementation of this initiative can occur through clearly defined phases.

• **Phase 1**: Conduct pilot projects in district hospitals.
• **Phase 2**: Integrate ECG devices into existing patient referral pathways.
• **Phase 3**: Expand nationally within broader cardiovascular disease programs.

**Figure 1:**
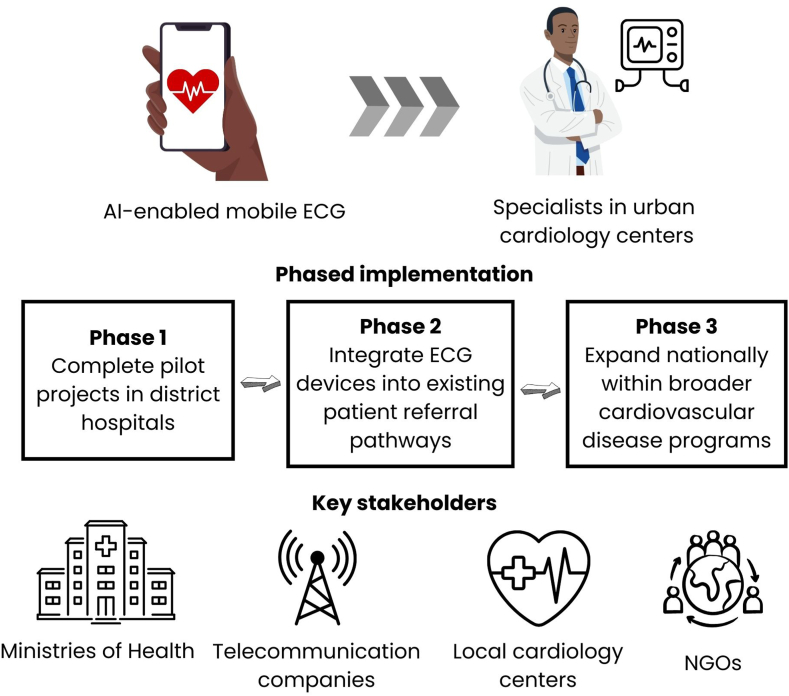
**AI-Enabled mobile ECG integration: phased implementation and key stakeholders.** AI, artificial intelligence; ECG, electrocardiogram; NGO, nongovernmental organization.

Key stakeholders include ministries of health (for policy and oversight), telecommunications companies (for reliable data transmission), local cardiology societies (for clinical training and support), and nongovernmental organizations to facilitate logistics and scaling efforts. This structured implementation directly addresses current gaps in diagnostic and referral capacities, significantly reducing prehospital delays and improving health care responsiveness^6^ (Figure 1).

## Addressing implementation challenges

### Digital infrastructure and electronic health record deficiencies

Limited digital infrastructure represents a significant barrier to successful AI implementation in SSA. Less than one-third of health care facilities have stable electricity or reliable internet access, and electronic health records (EHRs) are not widely available, resulting in fragmented patient data and reduced AI effectiveness.^3^^,^^7^ Addressing this issue requires strategic investment in digital health care infrastructure, such as solar-powered health information kiosks and cloud-based EHR systems compatible with offline use. These investments not only support immediate AI applications but also provide broader benefits to health care systems and long-term scalability.^8^

### Human resource constraints

SSA experiences severe shortages in specialized cardiology personnel, with <2 interventional cardiologists per 10 million inhabitants in many regions.^9^ However, AI enables effective task-shifting by empowering general health care workers and nurses through clinical decision-support tools.^10^ Developing regional AI training centers within academic hospitals, modeled after successful initiatives such as India’s Wipro GE Healthcare collaborations, could rapidly build local expertise. These centers would focus on practical training for health care providers in device usage, troubleshooting, and fundamental AI principles.^11^

### Regulatory and ethical frameworks

A lack of clear regulatory and ethical guidelines for medical AI in SSA creates uncertainty and reluctance among health care providers.^12^ Establishing harmonized regulatory frameworks through regional economic communities such as the Economic Community of West African States (ECOWAS) and the East African Community (EAC) can provide clarity regarding AI validation, clinical liability, and data protection standards. Leveraging frameworks like the African Union’s Digital Transformation Strategy could effectively standardize regulatory practices across the region.^13^

### Financial sustainability

Financial constraints, including high upfront costs for AI technology and competing funding priorities (such as malaria and HIV), pose significant challenges.^14^ Demonstrating the cost-effectiveness of AI interventions through targeted pilot projects focused on STEMI and heart failure management is essential. These studies should highlight potential reductions in transportation costs, unnecessary patient referrals, and long-term complications. Securing multilateral funding from entities such as the World Bank’s Global Financing Facility could help sustain these AI initiatives.^15^

### Integrating AI within primary health care systems

Critics often question the feasibility of implementing advanced AI solutions in regions where basic primary care services are already lacking. It is important to emphasize that AI and primary health care are complementary rather than competing priorities. AI applications, such as automated chatbots for medication adherence and vital sign monitoring, effectively extend the reach of health care workers, particularly in resource-constrained settings.^16^ Rather than replacing necessary systemic reforms, AI solutions can integrate effectively within existing health care frameworks to enhance overall service delivery.

## Roadmap for future action

To make AI sustainable in interventional cardiology across SSA, we need a focused and practical strategy. First, reliable and affordable EHR systems with offline access are essential to support data-driven AI tools in catheterization laboratories and emergency settings. Training must also be prioritized by integrating AI education into medical and nursing curricula, along with fellowships that focus on digital tools in interventional cardiology. These efforts will help build local expertise. AI models should be trained on regional data to reflect local patient profiles and reduce errors from using data developed elsewhere. Building high-quality cardiovascular datasets through local and cross-country collaborations is key. Clear and region-specific regulations must guide how AI is used in procedures, including standards for safety, data privacy, and ethical practice. Finally, AI tools should be built into national health plans for cardiology and backed by long-term funding so they can continue without relying on donors.

## Conclusion

Strategically applying AI solutions tailored to the specific challenges faced by SSA offers a significant opportunity to improve cardiovascular care equity and outcomes. Addressing core barriers through structured implementation steps can realistically transform the health care landscape, making cardiovascular services more accessible and responsive in resource-constrained regions.
